# Supporting PhD Students to Become Faculty in Gerontological Social Work: AGESW’s Fellowship Program

**DOI:** 10.1093/geroni/igaa057.1794

**Published:** 2020-12-16

**Authors:** Rebecca Mauldin, James Lubben

## Abstract

In the United States, the field of social work faces a critical shortage of students and faculty with expertise in gerontology needed to meet the growing needs of an aging society. To help recruit, train, and retain aging-related social work practitioners, researchers, and educators, the Association for Gerontological Education in Social Work (AGESW) created the Pre-Dissertation Fellowship Program in 2010. AGESW provides leadership in the areas of gerontological social work education, research, and policy and its PDFP was designed to support doctoral students in their education and future careers. In this 10th anniversary year of the PDFP, this symposium presents multiple perspectives of PDFP program evaluation. The first paper uses qualitative data from eight years of PDFP evaluations to identify types of professional skills attained through the program and areas of professional development missing from PDFP fellows’ home doctoral programs. The second paper uses quantitative data from a retrospective survey administered to PDFP alumni to describe their perspectives on the effects of the program. The third paper uses data from a retrospective survey of three cohorts of PDFP alumni to demonstrate the use of social network analysis for program evaluation. The fourth and final paper uses an idiographic approach to explain benefits of the PDFP from the perspectives of early stage scholars who participated in the program. Overall, the symposium provides evidence that suggests the effectiveness of the PDFP in building professional networks, mentoring doctoral students, and teaching academic skills and discusses using the PDFP model in other gerontological fields.

